# Engineering Toolbox for Systematic Design of PolyHIPE Architecture

**DOI:** 10.3390/polym13091479

**Published:** 2021-05-04

**Authors:** Prachi Dhavalikar, Jason Shenoi, Karim Salhadar, Malgorzata Chwatko, Gabriel Rodriguez-Rivera, Joy Cheshire, Reza Foudazi, Elizabeth Cosgriff-Hernandez

**Affiliations:** 1 Department of Biomedical Engineering, University of Texas, Austin, TX 78712, USA; prachisdhavalikar@gmail.com (P.D.); j.shenoi00@gmail.com (J.S.); karimsalhadar@gmail.com (K.S.); m.chwatko@uky.edu (M.C.); joycheshire@utexas.edu (J.C.); 2 Department of Chemical Engineering, University of Texas, Austin, TX 78712, USA; gabriel_rodriguez@utexas.edu; 3 Department of Chemical and Materials Engineering, New Mexico State University, Las Cruces, NM 88003, USA; rfoudazi@nmsu.edu

**Keywords:** polyHIPEs, emulsion stability, thermodynamics, pore architecture, emulsion viscosity, pore size

## Abstract

Polymerization of high internal phase emulsions (polyHIPEs) is a well-established method for the production of high porosity foams. Researchers are often regulated to using a time-intensive trial and error approach to achieve target pore architectures. In this work, we performed a systematic study to identify the relative effects of common emulsion parameters on pore architecture (mixing speed, surfactant concentration, organic phase viscosity, molecular hydrophobicity). Across different macromer chemistries, the largest magnitude of change in pore size was observed across surfactant concentration (~6 fold, 5–20 wt%), whereas changing mixing speeds (~4 fold, 500–2000 RPM) displayed a reduced effect. Furthermore, it was observed that organic phase viscosity had a marked effect on pore size (~4 fold, 6–170 cP) with no clear trend observed with molecular hydrophobicity in this range (logP = 1.9–4.4). The efficacy of 1,4-butanedithiol as a reactive diluent was demonstrated and provides a means to reduce organic phase viscosity and increase pore size without affecting polymer fraction of the resulting foam. Overall, this systematic study of the microarchitectural effects of these macromers and processing variables provides a framework for the rational design of polyHIPE architectures that can be used to accelerate design and meet application needs across many sectors.

## 1. Introduction

**Figure 1:**
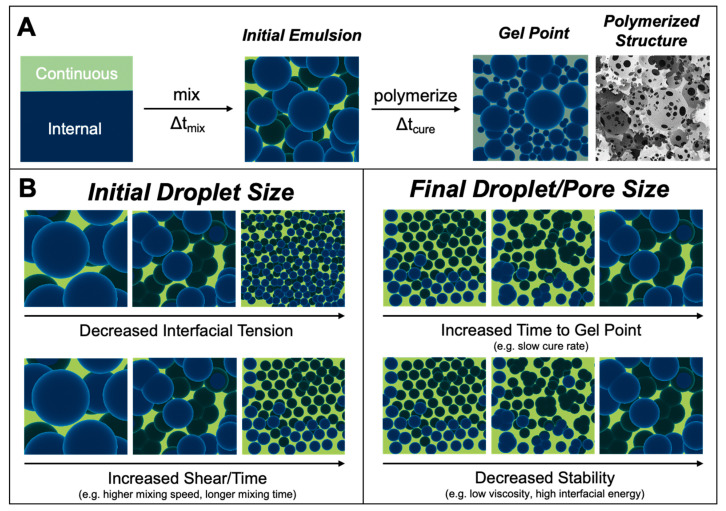
Generation of polyHIPE pore architecture based on emulsion templating parameters. (**A**) Schematic of emulsion fabrication: mixing of the two phases (duration of mixing is Δt_mix =_ Δt_Mf_ − Δt_Mi_, M_f_ = end of mixing and M_i_ = beginning of mixing), emulsion at gel point (time to gel point is Δt_cure =_ Δt_Mf_ − Δt_gel_, t_gel_ = gel point), and resulting architecture after crosslinking. (**B**) Overview of thermodynamic and polymerization parameters that affect polyHIPE architecture.

The use of porous polymeric materials has expanded to a wide variety of applications including sensors, solid phase synthesis, membrane processes, catalysis and biomedical applications [1,2,3]. Emulsion templating is a fabrication strategy that can be used to produce highly porous polymeric materials for these applications. A high internal phase emulsion (HIPE) is defined as an emulsion where the volume of the internal phase is greater than 74% of the continuous phase [4]. Subsequent polymerization of the continuous phase locks in the internal geometry of the dispersed droplets, producing a highly porous foam known as a polyHIPE [5,6]. The tunable nature of this system yields foams with a vast range of properties and diversity in applications. Given that the microarchitecture of polyHIPE foams is critical for functioning in many of the applications of interest, tuning pore architecture is a key area of study. Although research has elucidated key relationships between emulsion variables and foam architecture, researchers still often use trial-and-error to identify parameters that yield target pore architectures due to competing effects. This approach is both time intensive and costly. As such, there is great benefit in developing a more systematic approach to engineer polyHIPE architectures. We begin this analysis with a review of how HIPEs are used to generate foam pore architectures. The dispersed droplets in the emulsion template the pores in the resulting foam after the polymerization of the continuous phase (Figure 1). Researchers utilize emulsion science to control the droplet volume and size as a means of controlling the resulting foam pore size and porosity. Given that emulsions are only kinetically stable, the initial droplet size after emulsification will tend to increase in size due to coalescence and Ostwald ripening until the gel point of the continuous phase, which locks in the emulsion geometry. Thus, effects on polyHIPE microarchitecture can be divided into three key parameters during the emulsion-templating process: (1) initial droplet size; (2) emulsion stability; and (3) time to gelation.

The initial droplet size is dependent on both the interfacial energy and applied shear during emulsification. The total surface energy for a given interface is dependent on the surface energy of the two phases and the emulsifier (type, concentration) used to reduce the interfacial tension and act as a barrier between the phases. The free energy of the interface is a product of the interfacial tension and the surface area generated during the emulsification process. A reduction of droplet size with increasing surfactant concentration is typically observed in HIPEs, because the surfactant can enhance the stability of droplets during the emulsification. The emulsification process is a competition between droplet breakup and coalescence with higher surfactant content favoring the competition towards breakup. In addition, droplet breakup is controlled by the competition between shear forces and Laplace pressure, termed as dimensionless Capillary number. If the capillary number exceeds a critical value (which depends on the viscosity ratio of the dispersed phase to the medium), droplet breakup takes place [7]. It has been found that the higher surfactant coverage can decrease the critical capillary number [8]. Therefore, higher surfactant concentration facilitates the coverage of new formed interface and enhances the breakup, resulting in smaller droplet size after emulsification. Similarly, an increase in the work added to the system to generate new surface area in the form of mixing (homogenizer, ultrasound, overhead stirrer, microfluidics, duration of mixing) will increase the capillary number and generate smaller droplets [9,10,11]. The second parameter that plays a critical role in the emulsion template for pore architecture is the stability of the emulsion. Emulsions are energetically driven to reduce the surface area of the interface. Thus, the internal droplet size will increase over time as a function of droplet coalescence with the rate of phase separation dictated by the emulsion stability [12,13]. Increases in surface tension (i.e., higher Laplace pressure of droplets) due to greater disparities between the phases will typically result in a more rapid rate of droplet size increase prior to cure [14]. In addition, increased viscosity and some emulsifiers (e.g., nanoparticles) can slow down or prevent droplet coalescence. The final parameter is the amount of time that the emulsion can coarsen before the polymerization of the continuous phase locks in the emulsion geometry. Different modes of radical initiation (UV, redox, or thermal) and their respective chemistries can vary cure times. Longer cure times present opportunities for change in the internal geometry before the gel point is reached [15]. This fundamental framework of the drivers for polyHIPE architecture has been supported by numerous studies that have investigated one or two of these parameters [16,17,18]. However, it remains difficult to gauge the relative impact of each of these drivers without a cohesive study providing a direct comparison of individual and synergistic effects on polyHIPE architecture.

The objective of this study was to generate a framework for the rational design of polyHIPE architectures using a systematic study of the relative effects of emulsion variables on pore architecture. To this end, four different macromers were chosen to provide a design space for this evaluation: 1,4 butanediol diacrylate (BDDA), neopentyl glycol diacrylate (NGDA), 1,4 butanediol dimethacrylate (BDMA) and propylene fumarate dimethacrylate (PFDMA). The effect of surfactant concentration (5–20%), mixing speed (500–2000 RPM), macromer molecular hydrophobicity (logP, 1.9–4.4) and macromer viscosity (~6–170 cP) on the resulting polyHIPE architecture was characterized. The effect of viscosity on initial droplet size was studied. Then, toluene was used as a diluent to broaden the range of viscosities investigated and provide a means to delineate the effects of organic phase viscosity from molecular hydrophobicity. Finally, we investigated the use of a reactive diluent, 1,4-butanedithiol (BDT), to increase pore size by reducing organic phase viscosity without changing the resulting polymer volume fraction. Overall, this comprehensive evaluation provides additional insight into the rational design of polyHIPE architecture to accelerate design target attainment and meet application needs across many sectors.

## 2. Materials and Methods

### 2.1. Materials

Polyglycerol polyricinoleate 4125 (PGPR) was donated by Paalsgard. All other chemicals were purchased from Sigma Aldrich (St. Louis, MI, USA) unless otherwise noted. BDMA, NGDA, and BDDA were filtered through an aluminum oxide column to remove monomethyl ether hydroquinone inhibitor. The purified product was stored at 4 °C under a nitrogen blanket until use.

### 2.2. Molecular Hydrophobicity Estimates

Octanol–water partition coefficients (logP) were used as a measure of macromer hydrophobicity and were estimated based on molecular structures using Molsinspiration Chemoinformatics software (https://www.molinspiration.com, accessed on 3 May 2021). Estimates were based on the chemical structure of each compound as the sum of its nonoverlapping molecular fragments with group contributions obtained by fitting calculated logP with experimental logP for a training set of more than 12,000 molecules [19]. Adjusted logP values for mixtures was based on molar ratios of the mixture.

### 2.3. PFDMA Synthesis

PFDMA was synthesized in a two-step process detailed previously by Moglia et al. [16]. Briefly, propylene oxide was added dropwise in 2-butanone to a solution of fumaric acid and pyridine and refluxed at 75 °C to generate diester, bis (1,2 hydroxypropyl). Following purification, the diester was end-capped with methacrylate groups using methacrylol chloride in the presence of trimethylamine and purified.

### 2.4. Viscosity Measurements

Macromer and organic phase viscosity was measured using a Brookfield DV-E Viscometer. For BDDA, BDMA, and NGDA, 0.5 mL of macromer was loaded into the instrument and viscosity was tested at 100 RPM for 1 min. The viscosity for PFDMA was tested at 12 RPM for 1 min. For combinations of macromer and diluent, the two reagents were first mixed in a SpeedMixer at 2500 RPM for 2.5 min and then tested at 100 RPM for 1 min.

### 2.5. PolyHIPE Fabrication

Fast-curing redox polyHIPEs were fabricated utilizing a FlackTek SpeedMixer. For the organic phase, macromer and surfactant (5, 10, or 20 wt% PGPR) with either 1 wt% benzoyl peroxide (BPO) initiator or trimethylaniline (TMA) reducing agent were mixed at 2500 RPM in the FlackTek SpeedMixer for 2.5 min. After homogeneous mixing of the organic phase, an aqueous solution of calcium chloride (1 wt%) was added to the organic phase in three additions and mixed at 500 RPM for 2.5 min. The emulsion containing the initiator and the emulsion containing the reducing agent were then transferred into a double barrel syringe with a mixing head. The emulsions were injected at a 1:1 ratio into 2-mL microcentrifuge tubes and placed in a 37 °C aluminum bead bath overnight.

A similar procedure for polyHIPE fabrication as described above was followed to determine the effect of mixing speed on polyHIPE architecture. After mixing macromer, surfactant (5 wt%), and initiators at 2500 RPM for 2.5 min, an aqueous solution of calcium chloride (1 wt%) was added to the organic phase in three additions and mixed at varying speeds 500, 1000, or 2000 RPM for 2.5 min.

For diluent studies, the organic phase was added to a cup and the toluene (30, 50, or 70 wt%) or BDT (10%, 20%, or 30% molar ratio) was added with 5% PGPR along with 1 wt% BPO or 1 wt% TMA. The resulting mixture was then mixed at 2500 RPM in the SpeedMixer for 2.5 min prior to emulsification and crosslinking as described above. A complete matrix of the different HIPE formulations fabricated in these studies can be found in Supplemental Table S1.

### 2.6. Emulsion Rheology

Rheological properties of the HIPE formulations measured using a rheometer (Discovery HR-2 Hybrid) with a parallel plate geometry (Anton Paar Measuring Cone CP40, 40 mm diameter). Each HIPE formulation was pipetted (~0.5 mL) onto the plate at 25 °C and viscosity measurements were taken at shear rates between 0.1–100 s^−1^.

### 2.7. Stability Studies

Stability of BDDA, NGDA, BDMA, PFDMA, and poly(propylene glycol) dimethacrylate (PPGDMA) HIPEs was monitored over a period of five days. Briefly, HIPEs were prepared as described above without the addition of an initiator. Emulsions were loaded in 4 mL glass vials, tightly capped and stored at room temperature (25 °C) for the duration of the study. Photographs of emulsions were captured every hour for the first two hours after fabrication and then every 48 h.

### 2.8. Cure Time and Gel Fraction

PolyHIPE cure times were measured using a standard tack-free test. Briefly, HIPEs were gently pricked repeatedly with the sharp point of a spatula until no uncured HIPE was observed on the spatula tip and the time was noted as cure time. For gel fraction studies, polyHIPEs were sectioned using an Isomet bone saw into 1 mm sections. Sections were then dried for 48 h, weighed, and placed into glass vials with dichloromethane (DCM) at a ratio of 10 mg polyHIPE to 1 mL DCM on a shaker table at 60 RPM for 48 h. After extraction, DCM was decanted and specimens were placed into vacuum for 48 h at 70 °C. The dry mass was measured and gel fraction was calculated after correcting for surfactant concentration according to the following correction:

(1) $$Gel\;Fraction\;\left(\%\right)=\frac{Post\;Extraction\;\left(g\right)}{\frac{Pre\;Extraction\;\left(g\right)}{Surfactant\;Concentration\;\left(decimal\right)+1}}$$

### 2.9. Effect of Cure Time

HIPEs were fabricated and stored for varying durations (t = 2 min, 1 h, 4 h, and 24 h) prior to photopolymerization to delineate the role of initial droplet size from droplet coalescence prior to cure on resulting foam pore size. Briefly, HIPEs were fabricated as described above but with a photoinitiator, 1 wt% phenylbis(2,4,6-trimethylbenzoyl)phosphine oxide, rather than redox initiation. Following HIPE formation in the FlackTek SpeedMixer, emulsions were carefully stored in the dark and cured by UV light (365 nm) for 10 min at the selected time frames. Characterization of the resulting pore architecture provided the means to quantify the rate of droplet coalescence.

### 2.10. PolyHIPE Architecture

PolyHIPEs were dried under vacuum for 24 h to remove water prior to characterization of pore architecture. Scanning electron microscopy (SEM, Phenom Pro, Nanoscience Instruments) was used to characterize the average pore size of each composition. Circular specimens from three different polyHIPE samples were sectioned into quarters and were fractured at the center. The specimens were sputter coated with gold and imaged in a Rastor pattern. At each point, images were captured at 2000×, 1000×, 800×, 500×, and 250×. Pore size measurements were conducted by measuring the diameters of the first 10 pores that crossed the median of each 500× magnification micrograph. Average pore sizes for each polyHIPE composition are reported (*n* = 300). A statistical correction was applied to account for the non-perfect spherical pores, h^2^ = R^2^ − r^2^ where R is the void diameter’s equatorial value, r is the diameter value measured from the micrograph, and h is the distance from the center [12]. Average diameter values were multiplied by this correction factor, resulting in a more accurate description of the pore diameter.

### 2.11. Statistical Analysis

The data are displayed as mean and standard deviation for each composition. One-way Anova was performed to determine any statistically significant differences between compositions. All tests were carried out at a 95% confidence interval (*p* < 0.05).

## 3. Results and Discussion

**Figure 2:**
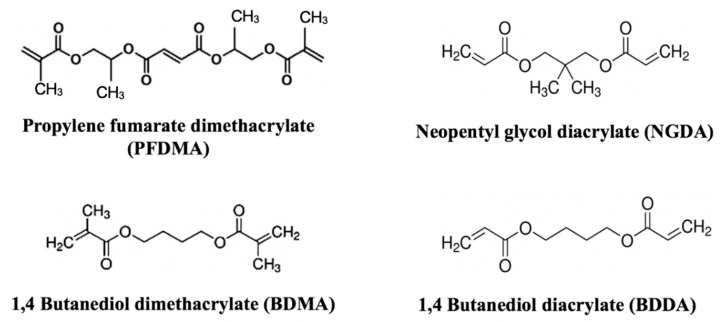
Molecular structures of PFDMA, NGDA, BDMA, and BDDA.

In this work, we selected four different macromers, a range of surfactant concentrations, and a range of mixing speeds to investigate the relative effect of key emulsion parameters on polyHIPE architecture. The macromer properties and chemical structures are presented in Table 1 and Figure 2. Collectively, these selections provide a broad design space to investigate key relationships between emulsion properties and the resulting polyHIPE architecture.

### 3.1. Effect of Surfactant Concentration

**Figure 3:**
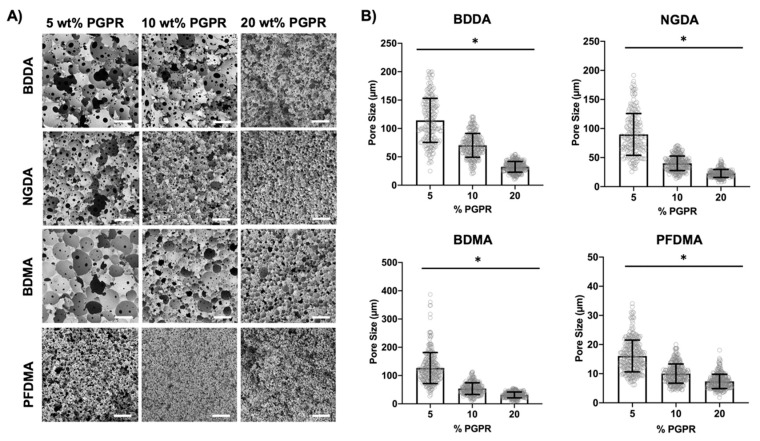
Representative scanning electron micrographs of fracture planes of BDDA, NGDA, BDMA, and PFDMA polyHIPEs fabricated with 5, 10, and 20 wt% surfactant (**A**) and the effect of increasing surfactant concentration on polyHIPE pore size for all four macromers (**B**). Scale bar is 100 μm. * denotes statistical significance, *p* < 0.05.

Across all four macromers, the polyHIPE pore size decreased with increasing surfactant concentration. For BDDA polyHIPEs the pore size dropped from ~100 µm to 40 µm, whereas NGDA, BDMA, and PFDMA polyHIPEs all displayed greater reductions in average pore size (80 µm to 20 µm (NGDA), 110 µm to 30 µm (BDMA), and 20 µm to 5 µm (PFDMA). All four polyHIPE samples were characterized by a narrower size distribution with high surfactant concentration (Figure 3B). It is interesting to note the differences in pore size averages and distribution within the macromers. NGDA, BDMA, and PFDMA all maintained very low pore size averages and narrower distributions compared to BDDA. We attributed this trend to potential differences in emulsion stability prior to gel point. Lastly, it was also observed that BDMA and PFDMA had a collapsed pore structure in some areas of the polyHIPE. We hypothesize that the pore structure was partially lost due to the high levels of PGPR. Potential differences in reaction kinetics between the methacrylate vs. acrylate chemistry, as confirmed from differences in cure time (Supplemental Table S2), may have rendered PFDMA and BDMA networks too slow to resist the collapse of the film due to the surfactant during polymerization. Similar phenomena of pore collapse at high concentrations of PGPR have been reported previously [20].

**Figure 4:**
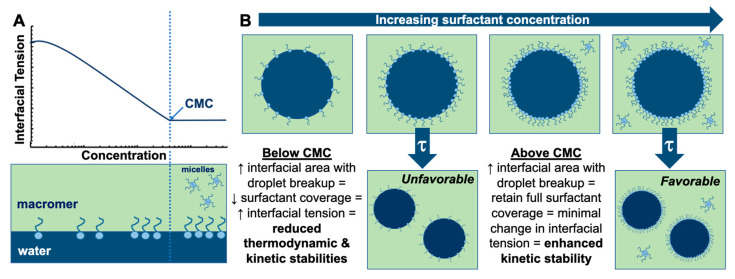
Overview of the role of surfactant concentration on droplet size in HIPEs. (**A**) Schematic representation of the decrease in interfacial tension with increasing surfactant adsorption at the interface until the CMC, after which interfacial tension is constant. (**B**) Schematic of the role of surfactant concentration on droplet breakup during emulsification above and below the CMC.

This trend of decreasing pore size with increasing surfactant concentration was attributed to enhanced droplet coverage during emulsification. Initial droplet formation and rate of phase separation are highly dependent on the thermodynamics of the system. The surface energy, product of the interfacial energy and surface area, increases significantly during emulsification [16]. This surface energy is often incorporated as reversible work during the process of emulsification and is a measure of the thermodynamic instability of the system that drives phase separation [4]. Surfactants are used to reduce the interfacial tension and form a barrier between the two phases. Interfacial tension will decrease with increasing surfactant adsorption at an interface until it reaches full surface coverage. Above the critical micelle concentration (CMC), the interfacial tension remains constant (Figure 4A). By increasing surfactant concentration in the HIPE system, the probability of stabilizing newly formed interfaces through surfactant adsorption increases, which favors droplet breakup, resulting in a decrease in droplet size (Figure 4B). There are two competing factors of HIPE stability as the surfactant concentration increases: (1) the higher interfacial energy of smaller droplet size induces higher driving force for coarsening through coalescence, (2) the narrower droplet size reduces the rate of Ostwald ripening. It follows that surfactants can be used to tune pore architecture by changing the initial droplet size and/or the rate of droplet coalescence. Studies investigating this effect have reported that an increase in surfactant concentration decreased the pore size [16,21]. Considering the general trend across the macromers, it can be concluded that surfactant concentration provides a robust route to tailoring polyHIPE pore size. Although these studies support previously established knowledge on the effect of surfactant on polyHIPE architecture, it is important to note how differences in the chemistries of the macromer may be affecting final pore structure and should be taken into consideration when investigating the use of surfactant to tune the pore size.

### 3.2. Effect of Mixing Speed

**Figure 5:**
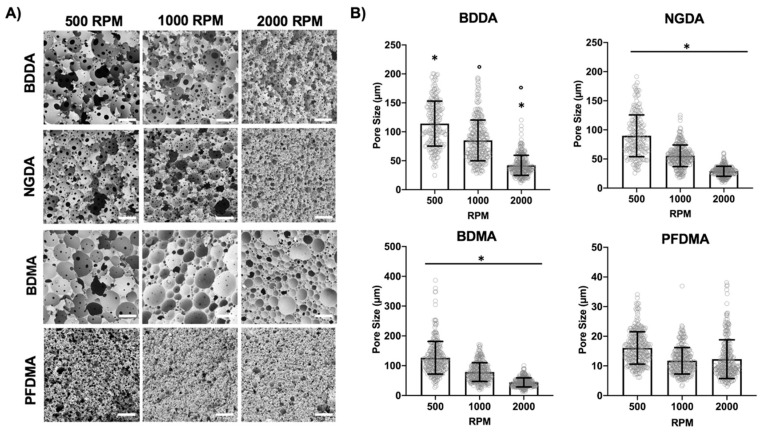
Representative scanning electron micrographs of fracture planes of BDDA, NGDA, BDMA, and PFDMA polyHIPEs fabricated at 500, 1000, and 2000 RPM (**A**) and the effect of increasing mixing speed on polyHIPE pore size for all four macromers (**B**). Scale bar is 100 μm. * and ° denotes statistical significance, *p* < 0.05.

Previous studies have demonstrated that increased mixing speed generally correlates with reduced pore size of the resulting polyHIPE and a reduction in pore size distribution [16,22,23]. In this study, all four macromers (5 wt% PGPR) maintained a similar trend in decreasing pore size with the increased mixing speed (Figure 5). The increase in mixing speed increases the shear stress applied on emulsion (and thus the capillary number), which was expected to increase droplet breakup. This reduction in size of the initial droplet size was then expected to result in smaller pore sizes of the resulting polyHIPE as described above [13,15]. It is interesting to note that the effect was much less pronounced for PFDMA than the other macromers. We have previously reported that mixing speed of PFDMA HIPEs at higher surfactant concentrations (10, 20 wt% PGPR) decreased in pore size and pore size distribution [16]. Based on the results, we are able to show that polyHIPE pore size can be tuned by varying surfactant concentration and emulsification speed to control initial droplet size. Although the results followed expected trends, we noted that within each parameter there were differences in the average pore size and distributions between the macromers. We attributed this to the intrinsic properties of the different macromers, specifically viscosity and interfacial tension. The goal of the next set of studies was to investigate what effects macromer viscosity and molecular hydrophobicity had on HIPE stability and subsequent polyHIPE pore architecture.

### 3.3. Effect of Macromer Chemistry

In addition to the surfactant effect on interfacial tension and emulsion stability, we investigated the relative effect of macromer selection on polyHIPE architecture. Both the initial droplet size and the rate of droplet coalescence are highly dependent on the interfacial energy of the system [24,25]. In the case of water-in-oil emulsions, it follows that a more hydrophobic macromer may result in higher interfacial tension between the continuous and aqueous phase when surfactant concentration is kept constant [26]. In this study, we assumed that macromer chemistry had minimal effect on the surfactant adsorption at the interface, which could have a confounding effect on interfacial tension, as discussed above. We utilized model predictions of the octanol-water partition coefficients (logP) as a means of comparing molecular hydrophobicity of the macromers. The logP values of the selected macromers can be found in Table 1. These macromers provided a similar range as most water-in-oil HIPE macromers with logP values in the range of 2–4 [16,17,21] and provided the test bed to determine if molecular hydrophobicity within this range impacts polyHIPE architecture.

**Figure 6:**
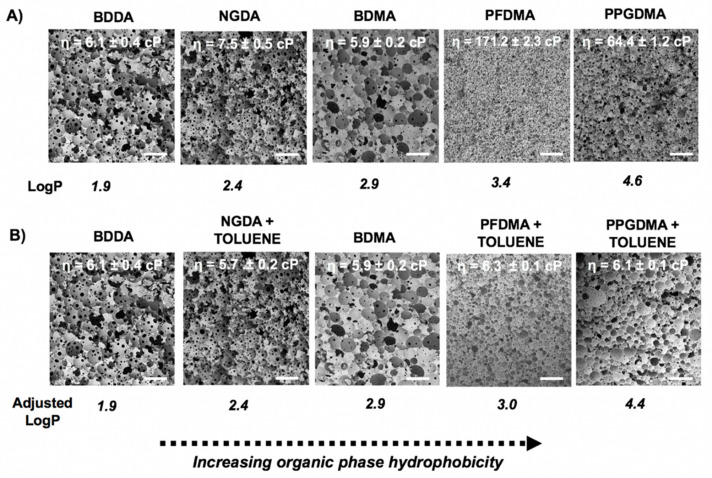
Representative scanning electron micrographs of fracture planes of BDDA, NGDA, BDMA, PFDMA, and PPGDMA polyHIPEs before (**A**) and after normalization of viscosity using toluene as diluent (**B**). LogP and adjusted logP values are reported below each SEM. Average organic phase viscosities are reported on each image. Scale bar is 100 μm.

For the purpose of this study, a fifth macromer, PPGDMA, was included in order to evaluate a broader range of macromer logP values. Macromers at lower logP values (BDDA, NGDA) displayed larger pore size as compared to the macromers with high logP (PPGDMA, PFDMA), Figure 6A. We hypothesized that increasing macromer hydrophobicity would increase initial droplet size due to an increase in interfacial tension and reduce Ostwald ripening with a corollary decrease in droplet coarsening. However, these more hydrophobic macromers also had a substantially higher viscosity. To eliminate convoluting effects of viscosity, macromers were diluted with toluene to comparable organic phase viscosities (Figure 6B). At comparable viscosities, there was not a strong trend with logP, which may indicate that the interfacial tension is dominated by the surfactant adsorption at the interface. Investigations into the effect of continuous phase hydrophobicity or interfacial tension on HIPE stability remains limited. One possible explanation is that there are confounding effects of molecular hydrophobicity and potential differences in adsorption behavior of PGPR at the interface for the different macromers. From literature, PGPR is established to be a robust surfactant, capable of stabilizing emulsions fabricated using food-grade oils that maintain very high logP values [27,28]. It was initially considered that at lower logP values, such as the case of BDDA, PGPR may have poor adsorption kinetics due to the continuous phase chemistry resulting in less stable emulsions. Deeper investigation comparing the logP values across macromers can rule out this theory. BDMA maintains a higher logP compared to NGDA or BDDA, therefore with better PGPR adsorption it should have maintained a smaller pore size and good stability. On the contrary, BDMA had a relatively large pore size distribution and was one of the first emulsions to show signs of phase separation in stability studies (Supplemental Figure S1). One potential limitation of this study is that interfacial tension was not directly measured and it would be informative to investigate how interfacial energy of each macromer changes with the addition PGPR [29]. From these results, we hypothesized that beyond the surfactant or macromer logP, the more significant driver for maintaining emulsion stability and potential indicator for pore architecture is the continuous phase viscosity. A continuous phase that has higher viscosity may be able to maintain stability even at high interfacial tension (e.g., PPGDMA) because the increased viscosity acts as a kinetic barrier to droplet coalescence. Based on these results, we concluded that it was not necessarily possible to draw direct connections between the continuous phase logP and how it can be harnessed to engineer pore architecture. It can likely be used as a guiding measure, but it is critical to understand that other factors such as viscosity, play be a bigger role in the resulting stability of the HIPE and as a result the pore architecture.

### 3.4. Effect of Emulsion Viscosity on Initial and Final Droplet Size

**Figure 7:**
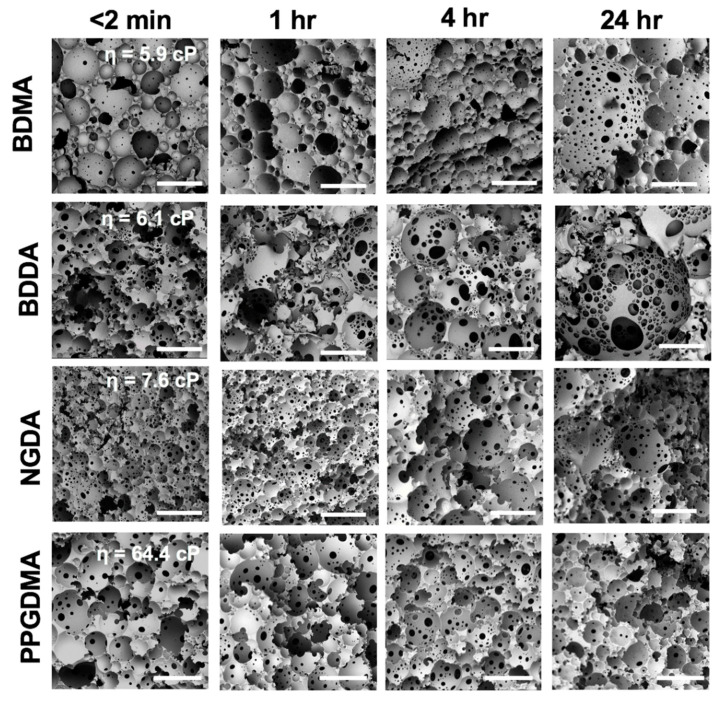
Representative scanning electron micrographs of fracture planes from BDMA, BDDA, NGDA, and PPGDMA polyHIPEs cured at specific time points. BDMA, BDDA, and NGDA polyHIPEs were imaged at 250× (300 μm scale bar). PPGDMA polyHIPEs were imaged at 1000× (100 μm scale bar).

To investigate the effect of emulsion viscosity on initial and final droplet size, the time to cure was controlled using photopolymerization. SEM analysis was used to quantify the change in pore size due to droplet coarsening and coalescence as a function of time until cure. The changes to the pore architecture from the initial droplet structure cured immediately after mixing (<2 min) up to 24 h prior to cure are shown in Figure 7. It was observed that polyHIPEs fabricated from low viscosity macromers, BDMA, BDDA, and NGDA, demonstrated an increase in pore size and distribution with increased time to cure; whereas the highly viscous PPGDMA polyHIPEs displayed minimal changes in pore size with increased time to cure (Figure 7 and Supplemental Figure S2). An increase in the viscosity of the continuous phase can act as a barrier to droplet coalescence, stabilizing the internal structure and limiting changes over time [17]. In contrast, the lower viscosity continuous phase allowed for droplet fusion with a corollary increase in pore size and pore size distribution. This increase in droplet size for the low viscosity macromer HIPEs was relatively slow relative to the rapid redox-initiated cure (<5 min) used in the previous experiments. Notably, there were minimal changes observed in the first hour after mixing, which suggests that the effect of macromer viscosity on pore size described above was due to differences in initial droplet size rather than coarsening or droplet coalescence. For these systems, the lower viscosity macromer HIPEs would only display differences in pore architectures due to coarsening or droplet coalescence if the cure rate was relatively slow (>1 h).

**Figure 8:**
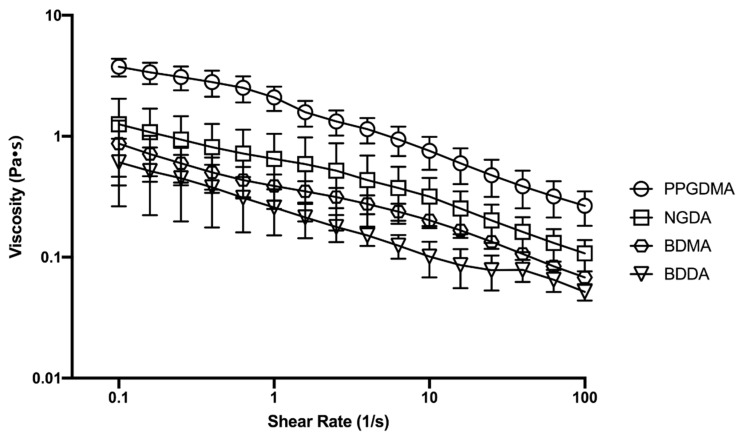
Log-log plot of relative viscosities of PPGDMA, NGDA, BDMA, and BDDA HIPEs at different shear rates. PPGDMA HIPEs maintained significant differences in viscosity (*p* < 0.05) at all shear rates compared to NGDA, BDMA, and BDDA HIPEs.

Based on this finding, we explored the cause for the observed decrease in initial droplet size with increased macromer viscosity. Droplet generation in concentrated emulsions under shear is a complex phenomenon determined by two processes: breakup and coalescence [30,31,32]. Droplet breakup is further governed by the critical capillary number, which depends on the viscosity ratio (λ and type of flow field (shear vs. elongation)) and is usually known as Grace curve [7]. While λ in the original Grace curve is the viscosity ratio of dispersed phase to continuous phase, when considering more concentrated emulsion systems, such as HIPEs, the λ has been modified as the viscosity ratio of dispersed phase to the emulsion [33]. In highly concentrated emulsions, droplets interact more frequently which can destabilize the droplets and result in more frequent breakup. Jansen et al. proposed that the breakup curve is expected to shift lower toward smaller capillary numbers as the volume fraction increases [33]. Therefore, Jansen et al. further proposed that droplet breakup in these systems should be predicted using a critical capillary number corrected with the relative emulsion viscosity (which is the ratio of emulsion viscosity to continuous phase viscosity). This proposal is supported with experimental and modeling data by Wieringa et al. who considered 80% oil-in-water emulsion in their studies [34]. The model predictions along with experimental data suggest that droplet breakup in highly concentrated emulsions is caused by average emulsion stress instead of the local stress of the continuous phase. This adjusted Grace curve proposed by Jansen et al. was used to interpret the effect of macromer viscosity on droplet breakup and initial droplet size in our system. To this end, emulsion viscosity for HIPEs fabricated with BDMA, BDDA, NGDA, and PPGDMA macromers was characterized at different shear rates, Figure 8. In a similar trend to macromer viscosities, PPGDMA HIPEs maintained the highest viscosity at all shear rates, followed by NGDA, BDDA, and BDMA.

Referring to the modified Grace curve, it can be noted that as emulsion viscosity increases, the modified viscosity ratio λ decreases, which is correlated with droplet breakup occurring at larger capillary numbers. In addition, modified critical capillary number increases with increasing emulsion viscosity, or in other words droplets break at lower shear rates. With higher viscosity emulsions, the associated shear stress is higher at a given shear rate, allowing for higher transmission of stress forces from the bulk to the drop interface [33]. Thus, we hypothesized that the higher shear stress resulted in greater droplet breakup in PPGDMA as compared to the lower viscosity emulsions [32].

This explanation likely only provides an explanation for one portion of droplet formation. Realistically, the generation of droplets in emulsions is not limited to simply breakup because the coalescence is also occurring, adding another level of complexity. The competition between breakup and coalescence is actively changing the droplet structure. Overall, the viscosity of continuous phase enhances the breakup process (since it increases the viscosity of HIPE) and slows down the coalescence. Collectively, this explains the correlation of increased pore sizes observed with decreased macromer viscosity.

Overall, these experimental observations highlight that continuous phase viscosity plays a critical role in dictating droplet breakup and coalescence for initial droplet size but can also impact rate of droplet coalescence after mixing. Thus, the role of viscosity must be considered in combination with the cure rate to predict the final effect on polyHIPE pore size. It is critical to understand how these competing mechanisms can be controlled in a target system in order to tune the initial droplet structure and tailor the cure rate to achieve target final pore sizes.

### 3.5. Diluent Effects on PolyHIPE Architecture

**Figure 9:**
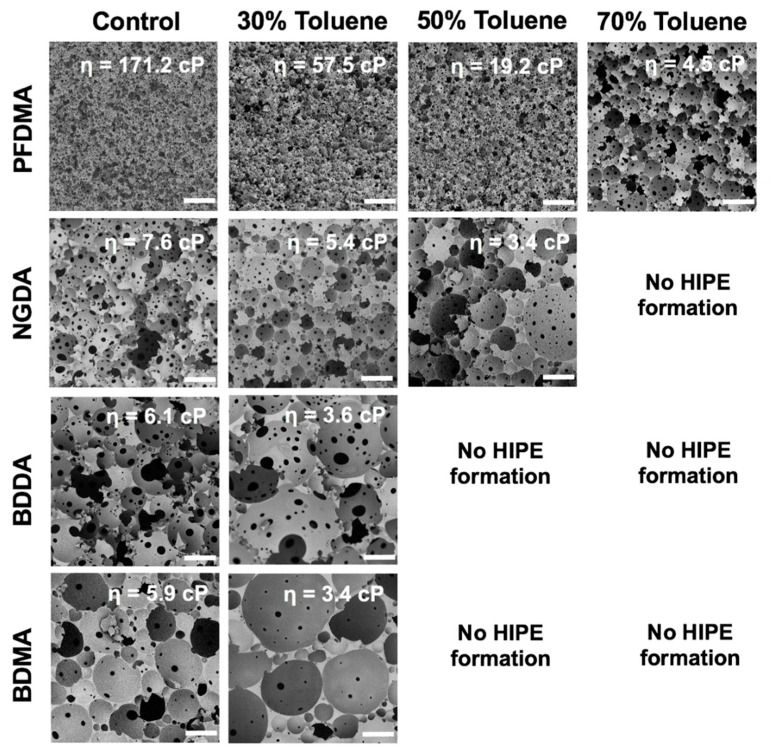
Representative scanning electron micrographs of polyHIPEs fracture planes illustrating the effect of lowering organic phase viscosity with increasing concentrations of toluene on pore architecture. Scale bar is 100 μm.

Given the strong effect of emulsion viscosity on polyHIPE architecture, we next investigated the utility of a diluent to increase pore size. Specifically, we investigated the effect of toluene diluent concentrations from 0 to 70 wt% of the organic phase for all four macromers. Diluents have been used to reduce the viscosity of the organic phase to allow for emulsification but diluents can also impact emulsion stability with a corollary effect on pore size [17]. As shown above, lower emulsion viscosity also corresponds to larger initial droplet sizes. The effect of diluent concentration on viscosity of the organic phase is listed in Table 2. Overall, increasing the toluene concentration from 0% to 70% resulted in a greater than 10-fold decrease in the viscosity of the PFDMA organic phase, which had the highest macromer viscosity. The other three macromers with lower starting macromer viscosities had a less pronounced effect of diluent concentration. The effect of this viscosity decrease on polyHIPE pore morphology is shown in Figure 9 with average pore size values reported in Table 2. Across the four macromers, as the organic phase viscosity decreased, the pore size increased albeit with different magnitudes. It was observed that PFDMA maintained about a 3-fold increase in pore size, but the average pore size remained small as compared to BDMA, NGDA, and BDDA. In contrast, BDDA, NGDA, and BDMA only displayed 1.5 to 2-fold increase in pore size. PFDMA was the only macromer for which stable HIPEs could be fabricated at all four selected toluene concentrations. This result suggests that a threshold viscosity, approximately 3.0 cP, was needed to enable formation of stable HIPEs without rapid phase separation.

The observed increase in pore size with reduced organic phase viscosity across all four macromers was attributed to reduced droplet breakup during mixing, as described above. A lower viscosity continuous phase can also increase mobility of the continuous phase and promote droplet coalescence. Beyond reduction in viscosity, the diluent can also influence the adsorption and arrangement of the surfactant at the interface with corollary effects on the interfacial tension. Regardless, these results demonstrate there is great promise in using continuous phase viscosity to modulate the polyHIPE pore size. The magnitude of pore size control is dependent on the starting macromer viscosity and is bounded by a threshold viscosities that can maintain HIPE stability until the gelation of the continuous phase locks in emulsion geometry. Again, this may be coupled with strategies that use polymerization chemistries or initiators that enable rapid curing [20,35].

### 3.6. Increasing Pore Size with a Reactive Diluent

As demonstrated in the previous study, using a diluent to reduce the organic phase viscosity can be used to increase pore size, especially with high viscosity macromers. However, this approach will reduce the polymer volume fraction in the resulting polyHIPE foam with corresponding loss of mechanical properties. We investigated the use of a co-monomer, BDT, as a reactive diluent that will reduce organic phase viscosity and react into the polymer network. This difunctional thiol compound can provide additional benefits for various applications. Methacrylate and acrylate chemistries are susceptible to premature termination under oxygen-rich environments. Under these conditions, high levels of initiating and propagating radicals are scavenged by ambient oxygen and converted to peroxy radicals rendering them unable to propagate monomer conversion [3]. This can affect foam properties including poor mechanical strength or leave high levels of leachable compounds. In contrast, with the use of the thiol-based compounds, the radicals abstract the hydrogen in the thiol, allowing the further propagation of the polymerization of the macromer, improving network properties [3].

In this study, BDT concentration from 0% to 30% (molar ratio) of the organic phase was utilized to evaluate the effect on HIPE formation and resulting polyHIPE architecture. Viscosity measurements demonstrated that BDT reduced the continuous phase viscosity for all macromers as compared to the control (Table 3). HIPE formation was confirmed at all BDT concentrations across the four macromers with no macroscopic phase separation observed prior to polymerization. Gel fraction for all samples tested was above 80% (Supplemental Table S2). Compared to control polyHIPEs, samples with BDT had a small increase in gel fraction. This confirmed that the BDT acted as a comonomer to react into the polymer network with all of the macromers tested. The small increase in gel fraction was attributed to the ability of BDT to increase monomer conversion as observed in studies conducted for thiol-based compounds [36].

**Figure 10:**
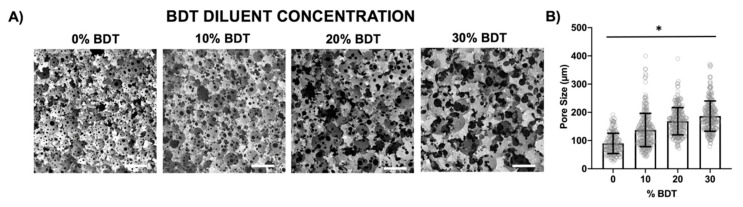
Representative scanning electron micrographs of fracture planes of NGDA polyHIPEs fabricated with 0, 10, 20, and 30 mol% reactive diluent concentration (**A**) and the effect of increasing reactive diluent concentration on polyHIPE pore size (**B**). Scale bar is 100 μm. * denotes statistical significance, *p* < 0.05.

NGDA was the only macromer that matched the trend of increasing pore sizes at higher concentrations of the reactive diluent (Figure 10). In addition, interconnects or windows between adjacent pores also increased in size with increasing BDT concentration. For the other three chemistries, there was a loss of the spherical pore architecture that characterizes polyHIPE foams (Supplemental Figure S3). Unlike toluene in the previous studies, the BDT can affect organic phase viscosity, polymerization kinetics, and network structure. Previous studies have investigated polymerization dynamics for thiol compounds and the reactivity to electron deficient bonds. Specifically looking at end groups that are present in the chemistries of the selected macromers, thiol-base reactivity follows: fumarate > diacrylate > methacrylate [37]. The fast reactivity with the fumarate bond coupled with significant tearing of the film at the interface may explain why PFDMA polyHIPEs lacked spherical pore structures. This can be applied to BDDA as well but, in this case, the effect was concentration dependent. In contrast, BDMA maintained a spherical pore structure as reactivity of the thiol with methacrylate end groups in BDMA may be relatively slower compared to the BDDA. Based on these studies, it can be concluded that the use of BDT as a diluent can be a promising method to tune the pore structure but careful consideration must be given to the fact that the reactivity of the compound can play a significant role in the resulting polyHIPE pore architecture.

### 3.7. Perspectives on the Rational Design of PolyHIPE Architecture

Modulation of polyHIPE architecture can result from tuning three key parameters—initial droplet size, time to gelation, and emulsion stability. Although there are a number of studies that investigate surfactant concentration and mixing within a system, this comparative analysis provides additional insight in how effective these strategies are across different polyHIPE chemistries. Our studies demonstrated that the most impactful strategy to tune pore size within a HIPE system was to modulate surfactant concentration. Based on the different averages across macromers, we observed that there was a 6-fold difference in pore size between the lowest and highest surfactant concentration (Table 4). This aligns well with previous studies that increasing surfactant concentration promotes HIPE stabilization and smaller pore sizes and more narrow pore size distributions [16,21]. Despite the diverse range of pore sizes that can be achieved with this methodology, it is important to consider this may not be an ideal method to investigate for specific polyHIPE applications such as tissue engineering, as high concentrations of surfactant can affect scaffold biocompatibility and cell-material interactions [38]. Further, depending upon the type of surfactant and macromer chemistry that is used, it may impart structural changes that can affect mechanical properties. The second parameter that can affect initial droplet size is the amount of work applied during emulsification that can be controlled with the mixing method or duration. From these studies, we observed a 4-fold decrease in pore size when the mixing speed was increased from 500 to 2000 RPM across the various macromers. This was attributed to increased droplet shear that can reduce initial droplet size. However, we did not see a large effect with the higher viscosity PFDMA macromer with low surfactant concentration. This may indicate that mixing speed may be a less universal method to tune pore architecture than surfactant concentration.

In addition, we investigated macromer hydrophobicity and viscosity effects on polyHIPE architectures. This investigation provides insight that will facilitate *a priori* selection of macromers for target polyHIPE architectures. We were able to provide a potential explanation of how viscosity affects initial droplet generation and further demonstrated how controlling the time till polymerization can allow for viscosity to affect the initial geometry. These studies suggested that cure rate of the HIPEs is a secondary entity that can be tuned to a certain extent to exert control on how intrinsic properties and fabrication parameters affect pore size. We further modulated the viscosity and it was observed that by tuning the continuous phase viscosity using toluene, pore size increased 4-fold as the viscosity was reduced but only for macromers with high viscosity. The effect on pore size for lower viscosity macromers was bounded by a threshold viscosity needed for HIPE formation and stability (~3 cP). These results are consistent with trends in the literature and confirm that reducing the continuous phase viscosity promotes droplet coalescence, a main driver of phase separation and emulsion destabilization. Our studies suggest that macromer viscosity may be a larger indicator of emulsion stability as compared to macromer hydrophobicity. Although there were differences between polyHIPEs of different macromer hydrophobicities, there was no clear trend within the range that permitted HIPE formation and stability (logP ~1.9–4.4). We observed that, even in situations where the interfacial tension was anticipated to be high, such as the macromers that maintain high logP values, higher viscosity of the macromer (and resulting HIPE) prevented destabilization. On the contrary, low viscosity of macromers such as BDMA or BDDA drove earlier phase separation, even in instances where the interfacial tension was hypothesized to be lower. It is important to note these last considerations are more applicable to surfactant-stabilized HIPEs. There are alternative techniques that have been pursed to stabilize emulsions, namely the use of nanoparticles. In these Pickering emulsions, nanoparticles adsorb at the interface between the two phases and create a physical barrier to droplet coalescence [23,39,40]. Although Pickering emulsions offer the ultimate resistance to coalescence and therefore are highly advantageous for certain applications, there are tradeoffs to consider such as closed-pore structure, which can significantly affect performance of the polyHIPEs depending upon the application [23].

## 4. Conclusions

This collective work provides insight into the relative effects of multiple parameters on polyHIPE architecture using a systematic study across several macromer chemistries. The dominant effect on pore size was observed with increasing surfactant concentration (~6 fold), whereas changing mixing speeds (~4 fold) displayed a reduced effect. Furthermore, it was observed that organic phase viscosity had a marked effect on initial droplet size and final pore size (~4 fold) with no clear trend observed with molecular hydrophobicity in this range (logP = 1.9–4.4). The efficacy of 1,4-butanedithiol as a reactive diluent was demonstrated and provides a means to reduce organic phase viscosity and increase pore size without affecting polymer fraction of the resulting foam. Overall, this systematic study of the microarchitectural effects of these macromer and processing variables provides a framework for the rational design of polyHIPE architectures. This engineering toolbox for the selecting parameters to tune polyHIPE architecture can be used to accelerate design and meet application needs across many sectors.

## Figures and Tables

**Table 1:** Physical and chemical properties of PFDMA, NGDA, BDMA, and BDDA.

Macromer	LogP	Viscosity (cP)	Density (g/mL)	Molecular Weight (g/mol)
PFDMA	3.4	171.2 ± 2.3	1.00	362
NGDA	2.5	7.6 ± 0.5	1.03	212
BDMA	2.9	5.9 ± 0.2	1.02	226
BDDA	1.9	6.1 ± 0.4	1.05	196

**Table 2:** Macromer logP *, viscosities at varying concentrations of toluene (logP = 2.4), adjusted logP values **, and associated pore sizes. (Not determined = n.d.).

Macromer	LogP *	[Toluene] (wt%)	Adjusted LogP **	Viscosity (cP)	Average Pore Diameter (μm)
PFDMA	3.4	0	3.4	171.2 ± 2.3	14 ± 5
		30	3.1	57.5 ± 1.3	18 ± 8
		50	2.9	19.2 ± 2.8	26 ± 8
		70	2.7	4.5 ± 0.1	50 ± 30
NGDA	2.5	0	2.5	7.6 ± 0.5	58 ± 18
		30	2.4	5.4 ± 0.1	78 ± 15
		50	2.4	3.4 ± 0.1	101 ± 76
		70	2.3	n.d.	n.d.
BDDA	1.9	0	1.9	6.1 ± 0.4	106 ± 36
		30	2	3.6 ± 0.2	142 ± 67
		50	2.1	n.d.	n.d.
		70	2.2	n.d.	n.d.
BDMA	2.9	0	2.9	5.9 ± 0.2	102 ± 37
		30	2.7	3.4 ± 0.1	141 ± 66
		50	2.6	n.d.	n.d.
		70	2.5	n.d.	n.d.

* Octanol–water diffusion coefficient calculated with the Molinspiration miLogP model based on molecular structures. ** Adjusted based on molar ratios of constituents.

**Table 3:** Organic phase viscosity of PFDMA, NGDA, BDDA, and BDMA diluted with BDT at varying concentrations. Viscosity values at 30% BDT for NGDA, BDMA and BDDA were not measurable. (Not determined = n.d.).

Macromer	LogP *	[BDT] (mol%)	Adjusted LogP **	Viscosity (cP)
PFDMA	3.4	0	3.4	171.0 ± 0.1
		10	3.2	168.3 ± 0.5
		20	3.0	149.5 ± 1.4
		30	2.8	130 ± 0.4
NGDA	2.5	0	2.5	7.6 ± 0.5
		10	2.4	6.9 ± 0.1
		20	2.3	6.4 ± 0.1
		30	2.2	n.d.
BDDA	1.9	0	1.9	6.1 ± 0.4
		10	1.8	4.9 ± 0.2
		20	1.8	4.4 ± 0.1
		30	1.8	n.d.
BDMA	2.9	0	2.9	5.9 ± 0.2
		10	2.8	5.3 ± 0.1
		20	2.5	4.3 ± 0.1
		30	2.5	n.d.

* Octanol–water diffusion coefficient calculated with the Molinspiration miLogP model based on molecular structures. ** Adjusted based on molar ratios of constituents.

**Table 4:** Observed polyHIPE pore size ranges for various HIPE fabrication parameters including mixing speed, surfactant concentration, and viscosity.

Parameter	Range for Parameter	Range of PolyHIPE Pore Size	Fold Change (Pore Size)
Mixing speed	500–1000 RPM	30–120 μm	4×
Surfactant concentration	5–20% (wt%)	20–120 μm	6×
Viscosity	6–170 cP	20–80 μm, 50–200 μm	3–4×

## Data Availability

Data supporting reported results is available from the corresponding author upon request.

## Supplementary Materials

The following are available online at https://www.mdpi.com/article/10.3390/polym13091479/s1, Table S1: HIPE Matrix for Formulations, Table S2: Gel Fraction and Cure Time, Figure S1: HIPE Stability, Figure S2: Effect of BDT Concentration on PolyHIPE Pore Size, and Figure S3: Time Course Study of Effects of Macromer Viscosity on PolyHIPE Pore Size.
