# Supplementary material for: Engineering Toolbox for Systematic Design of PolyHIPE Architecture
Source: Polymers (Basel). 2021 May 4;13(9):1479. doi: 10.3390/polym13091479 (PMC8124597; doi:10.3390/polym13091479)
Supplement: Supplementary file 1 [file polymers-13-01479-s001.zip › polymers-1200807-supplementary.pdf]

Supplemental Figures:

Supplemental Table 1: Composition matrix of HIPEs fabricated at different parameters for each of the four macromers PFMDA, NGDA, BDDA, and BDMA. (Not applicable = n.a.)

Macromer	Surfactant Concentration (wt %)	Mixing Speed	Toluene Diluent (mol %)	BDT Diluent (mol %)
PFDMA	5	500	0	0
	10	1000	30	10
	20	2000	50	20
			70	30
NGDA	5	500	0	0
	10	1000	30	10
	20	2000	50	20
			n.a.	30
BDDA	5	500	0	0
	10	1000	30	10
	20	2000	n.a.	20
			n.a.	30
BDMA	5	500	0	0
	10	1000	30	10
	20	2000	n.a.	20
			n.a.	30

Supplemental Table 2: Gel fraction and cure time for BDDA, NGDA, BDMA and PFDMA polyHIPEs fabricated at increasing mixing speeds, increasing surfactant concentrations, and increasing diluent concentrations.

Macromer	Composition	Gel Fraction (%)	Cure Time (Mins)
BDDA	500 RPM	97 ± 2	2.5
	1000 RPM	99 ± 3	2.5
	2000 RPM	94 ± 5	2.2
	5% PGPR	98 ± 2	2.5
	10% PGPR	99 ± 1	2.5
	20% PGPR	97 ± 2	2.5
	10 % BDT	99 ± 1	2
	20% BDT	98 ± 2	1.5
	30% BDT	99 ± 1	< 1
	30% Toluene	96 ± 2	3
NGDA	500 RPM	93 ± 1	2.5
	1000 RPM	99 ± 1	2.5
	2000 RPM	99 ± 1	2.7
	5% PGPR	98 ± 1	2.5
	10% PGPR	97 ± 1	2.6
	20% PGPR	98 ± 3	2.5
	10 % BDT	97 ± 1	2
	20% BDT	98 ± 1	1.3
	30% BDT	99 ± 1	< 1
	30% Toluene	92 ± 2	2.7
	50% Toluene	93 ± 3	2.6
BDMA	500 RPM	94 ± 3	4
	1000 RPM	95 ± 2	3.5

	2000 RPM	93 ± 1	3
	5% PGPR	95 ± 2	4
	10% PGPR	95 ± 1	4
	20% PGPR	86 ± 4	3.8
	10 % BDT	95 ± 2	3
	20% BDT	96 ± 2	2.5
	30% BDT	96 ± 4	2.5
	30% Toluene	93 ± 2	3
PFDMA	500 RPM	86 ± 3	6
	1000 RPM	85 ± 1	6.5
	2000 RPM	92 ± 1	6.5
	5% PGPR	86 ± 2	5
	10% PGPR	87 ± 3	5.7
	20% PGPR	83 ± 2	5.6
	10 % BDT	92 ± 3	4
	20% BDT	94 ± 2	2.5
	30% BDT	97 ± 1	2.5
	30% Toluene	88 ± 3	4.5
	50% Toluene	84 ± 3	4.3
	70% Toluene	86 ± 3	4

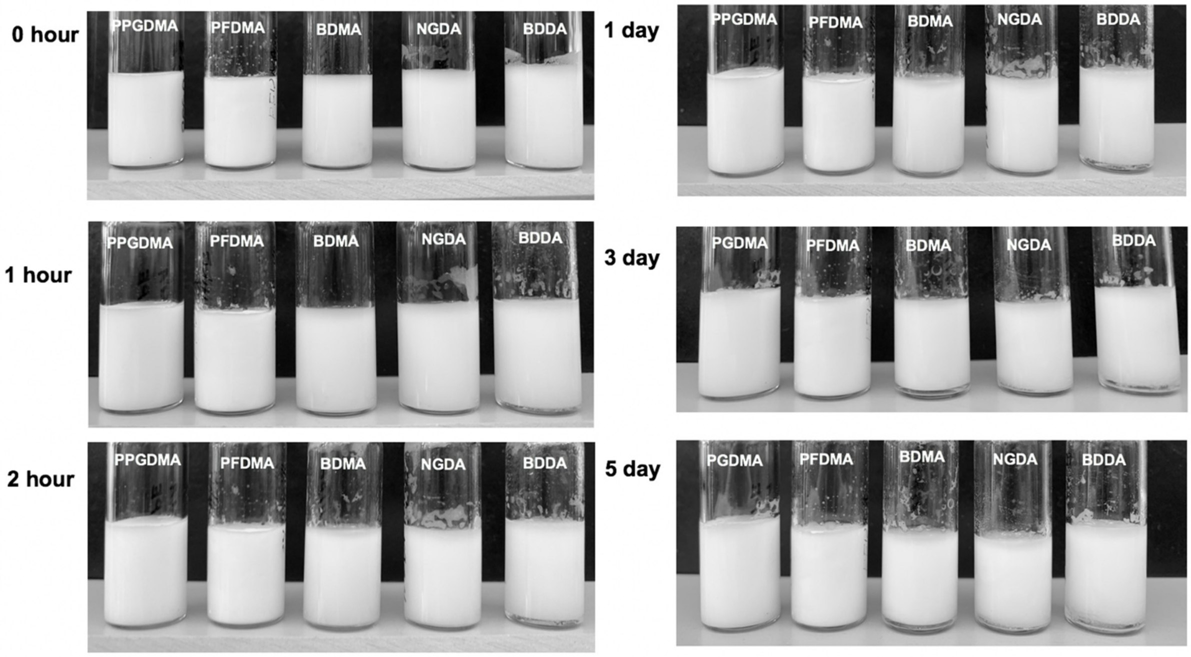

Supplemental Figure 1: Photographs of HIPEs fabricated for PPGDMA, PFDMA, BDMA, NGDA, and BDDA at various time points. Phase separation can be observed at the bottom of the glass vials starting at the 1 hour time point.

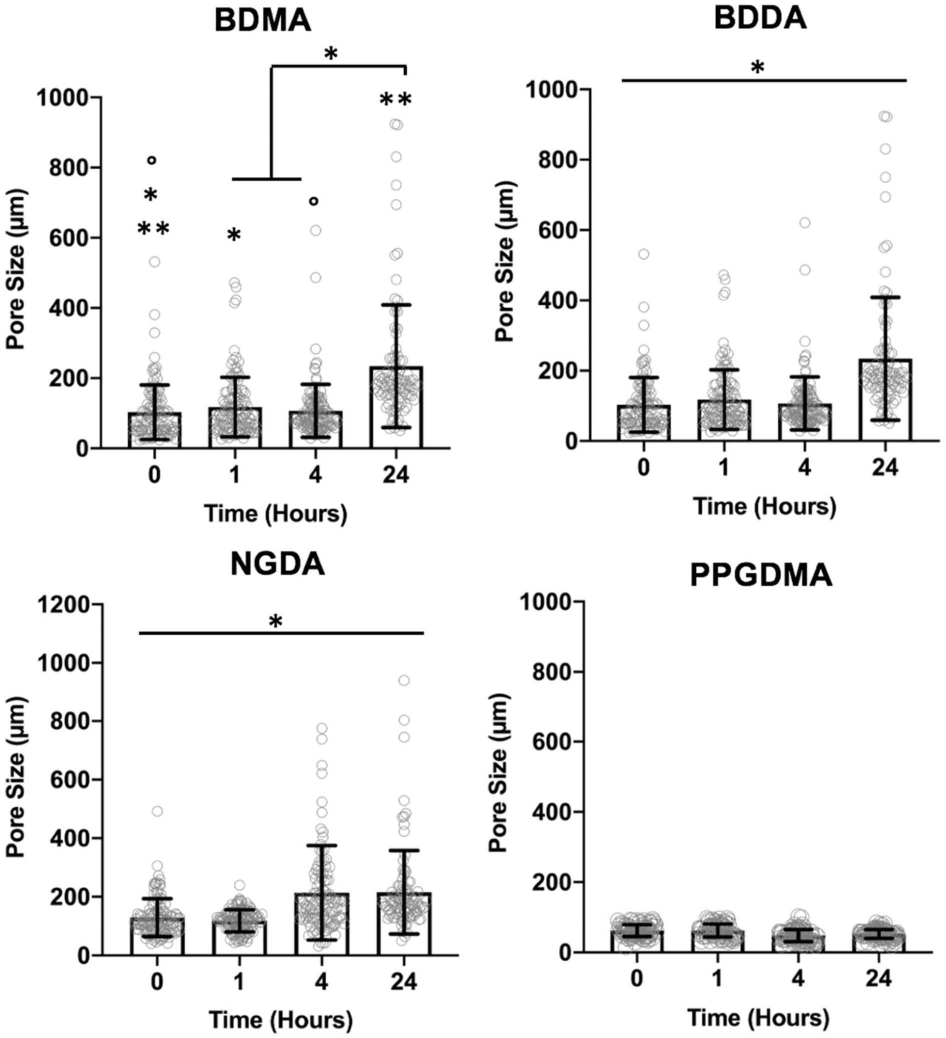

Supplemental Figure 2: Pore size distributions to evaluate changes in initial droplet size (time 0 on graphs corresponded to <2 min timepoint in SEM). Scale bar is 300 µm. *, °, and ** denotes statistical significance, $p<0.05$.

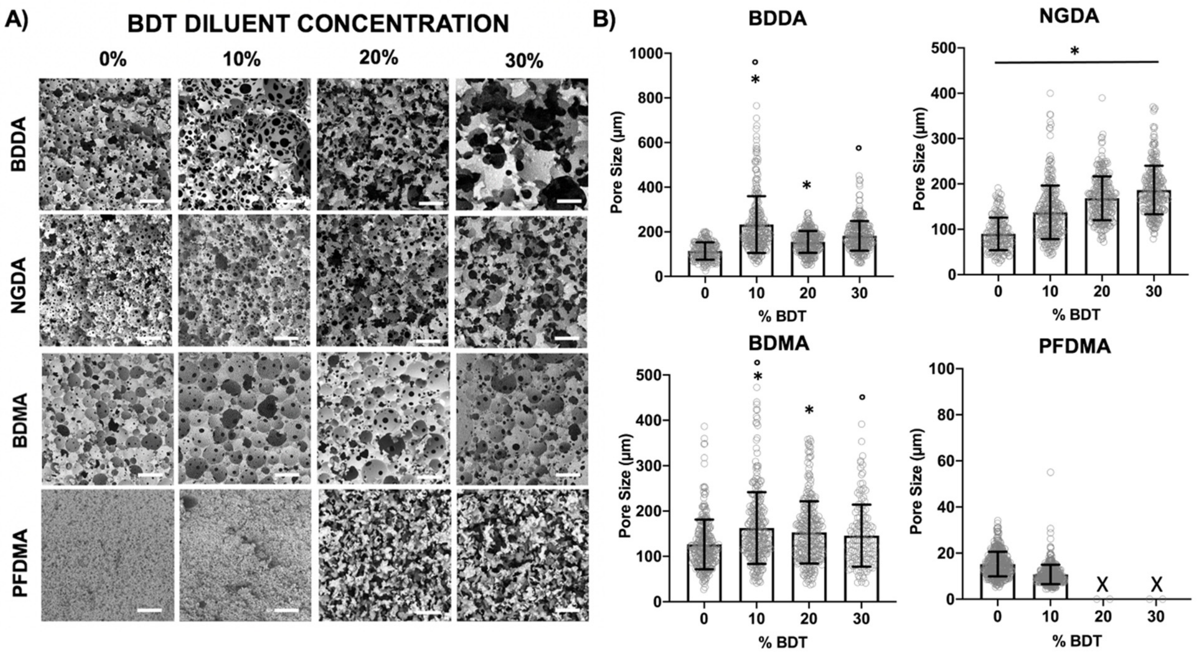

Supplemental Figure 3: Scanning electron micrographs of fracture planes of BDDA, NGDA, BDMA, and PFDMA polyHIPEs fabricated with 0, 10, 20, and 30 mol % reactive diluent concentration (A) and the effect of increasing reactive diluent concentration on polyHIPE pore size (B). Scale bar is 300 μm. * and ° denotes statistical significance, $p<0.05$.
